# Big Data analysis to improve care for people living with serious illness: The potential to use new emerging technology in palliative care

**DOI:** 10.1177/0269216317726250

**Published:** 2017-08-14

**Authors:** Amara Callistus Nwosu, Brendan Collins, Stephen Mason

**Affiliations:** 1 Marie Curie Palliative Care Institute Liverpool (MCPCIL), University of Liverpool, Liverpool, UK; 2 Department of Public Health and Policy, University of Liverpool, Liverpool, UK

We read with interest the editorial by Peter Tanuesputro,^1^ which describes how Big Data analysis of linked data has the potential to support palliative care by improving identification of patient needs. The editorial described how the use of predictive algorithms (built using routinely collected data) could help to personalise care for people with palliative care needs. However, it is necessary to address the potential barriers that may prevent the full potential of Big Data from being realised.^2^ Some of these challenges (highlighted in the editorial) include the inadequate capture of certain information, such as non-hospital and mortality data, patient experience information and available familial/social support. In addition to these challenges, we discuss five additional issues that need to be considered in order to make meaningful use of Big Data analysis in palliative care.

## The expertise challenge

Healthcare professionals are generally unaware of how Big Data can be used to improve palliative care delivery. Furthermore, there is a lack of collaborative multi-professional groups with expertise in key areas (such as Information Technology (IT), clinical practice, computer science, economics, statistics and research methods).^3^ A lack of expertise in the development, maintenance and analysis of electronic health record (EHR) systems may prevent adequate design of systems for the user. This may limit the quality of data collection and extraction (necessary for meaningful data analysis). Engagement with healthcare professionals is essential to support the design of digital systems that are necessary to improve the ability of clinical staff to work effectively. Additionally, data analysts are needed to make sense of the data generated by this process.^4,5^ Therefore, in order to realise the potential of Big Data, it is important to develop multi-professional groups with the expertise to use data meaningfully, to influence healthcare policy and clinical care delivery.

## The research methodology challenge

Big Data analysis has the potential to evaluate care in real time using techniques such as Machine Learning (a type of Artificial Intelligence (AI) that allows software applications to become more accurate in predicting outcomes without being explicitly programmed^6^), which can support quality improvement techniques in clinical practice.^7,8^ Machine Learning image processing has been used for accurate diagnosis of skin melanoma from photographs taken on a mobile device,^9^ and to estimate prognosis of patients with non-small lung cancer through analysis of microscopic pathology images.^10^ Consequently, developments of this technology, combined with the increasing use of linked integrated EHRs (e.g. the Kent Integrated Data Set^11^ and Kaiser Permanente’s HealthConnect system^12^), will potentially enable treatments to be evaluated in real time. For example, propensity score matching (a statistical matching technique that attempts to estimate the effect of an intervention by accounting for the covariates that predict receiving the treatment^13^) could use EHR data of an individual to estimate their likely response to a particular treatment.

Despite its potential, these techniques have not been exploited due to a lack of understanding and familiarity with Machine Learning and Big Data analysis. When clinical care has been evaluated using this approach, the necessary interventions (to improve or maintain care quality) will need to be determined. It is therefore important that research studies evaluate how on-going analysis of linked data can be used to improve care delivery. This creates methodological and ethical considerations for future research. For example, it may be possible to determine the effect of an intervention at any point of time during a prospective study. This presents the opportunity for innovative methodology, and also creates challenges of how to appropriately manage data during research studies.^14^ It may be more difficult for Big Data–driven studies to obtain ethical approval when the ‘research question’ is not simple or may emerge over time.

## The interoperability challenge

Poor interoperability between electronic healthcare record systems limits the ability to conduct meaningful Big Data analysis.^15^ It is important that future EHRs are designed to overcome inoperability (e.g. by adoption of Fast Healthcare Interoperability Resources (FHIR) standard protocol^16^) and to enable integration of third-party software applications to enable flexibility and customisability of EHR systems.^17^ Improved interoperability will improve the ability to capture and share data through enabling developers to create software to achieve specific functions. This could offer the ability to integrate app-based data collection processes, which may create the potential to record self-reported patient experience data.^18^ Many obstacles affecting meaningful health information exchange are political and economic; therefore, a careful digital strategy is necessary for successful healthcare reform. This strategy should ensure that developed technology is optimised for the needs of the user (e.g. healthcare professionals and patients). Furthermore, organisations must accept the need for technical and adaptive changes in order to optimise systems for workforce efficiency. The benefits from digital innovation are not just financial, and may include reconfiguration of service delivery and reimagining work.^3^ Failure to acknowledge these issues has led to high-profile failures, such as the UK National Programme for Information Technology (NPfIT – the ambitious programme to digitise secondary care which was launched in 2002, but closed in 2011). Analyses of NPfIT failure describe how the programme was too ambitious, too centralised and failed to adequately engage with organisations and staff.^3,19^ Data security and privacy are essential; if citizens are concerned about their data being shared, then this may cause these programmes to fail, such as NHS care.data in England.^20,21^ Therefore, it is essential that there is adequate engagement among political leaders, healthcare providers, clinicians and the technological industry, in order to overcome these barriers.^22^

## The challenge posed by other sources of Big Data

Large amounts of data are continually being generated by a growing number of sensor-based technologies (known as the Internet of Things (IoT)), which provide health-related data about individuals. This technology has the potential to provide useful data about an individual’s daily routine, which can personalise care according to a patient’s individual circumstances.^12^ Currently, much of this data are collected by wearable devices, which may be useful for individuals, but may not meet clinical standards of accuracy. In the future, if living environments are linked up to the IoT, then the scope of data collection may evolve beyond typically including sleep, movement and heart rate, to include eating and drinking habits and time spent on different types of activities. Further research is needed to determine how this data can be effectively used (in conjunction with linked data) to support care, and establish core outcomes for research using these methods.

## The challenge of treating the patient as a person despite developments of this technology

Although predictive algorithms aim to personalise care, there is a risk that this technology may be dehumanising if used poorly. Clinical decisions informed by data analytics alone could potentially lead to clinicians treating ‘the data’ rather than the individual. There is also a possibility that aspects of care lacking data (e.g. patient experience) will receive less focus than other areas with more data (e.g. mortality data). Consequently, it is important that data are primarily used to help inform treatment decisions for individuals (with acknowledgement of their personal circumstances and preferences) as opposed to a method of automating treatment decisions.

## Conclusion

The use of Big Data analysis presents a unique opportunity to identify palliative care needs to inform population and individual-level interventions. Future research is essential to address the challenges faced by Big Data analysis in palliative care, in order to determine how this technology can be meaningfully used to improve patient care.
